# A cohort study of medication adherence among patients with chronic obstructive pulmonary disease in Egypt

**DOI:** 10.1038/s41533-020-0188-9

**Published:** 2020-07-14

**Authors:** Joël Ladner, Mahinour El Badrawy, Anas Nofal, Joseph Saba, Etienne Audureau

**Affiliations:** 1 Epidemiology and Health Promotion Department, Rouen University Hospital, Rouen, France; 2 Axios International, Paris, France; 3 Assistance Publique Hôpitaux de Paris, Hôpital Henri Mondor, Public Health, Paris Est Université, Créteil, France

**Keywords:** Epidemiology, Patient education

## Abstract

Chronic obstructive pulmonary disease (COPD), the most common chronic respiratory disease, is expected to become the third leading cause of death worldwide in 2020. A prospective cohort study conducted in 2017 and 2018 aimed to identify factors associated with inhaler treatment adherence in patients with COPD in Cairo (Egypt). Physicians collected data regarding patient deaths, treatment-related adverse events, and patients’ social support (no support, patient, support by spouse, children, and siblings) from their patients with COPD. The reason for treatment discontinuation was categorized as per patient decision or per physician decision. Adherence was categorized as treatment continued or treatment stopped. Patients who decided to stop treatment were considered non-adherent to COPD therapy. A total of 1311 patients as well as 98 physicians and 205 pharmacists were included. Pharmacists and social support (spouse, children/siblings) were identified as key positive factors in patients’ decisions to adhere to their prescribed COPD treatment regimens. A total of 631 patients (48.1%) stopped the treatment, including 170 (27.0%) due to patient decision and 55 (8.7%) deceased. After Cox model analysis, a low number of patients (6–19) attended by the pharmacist was a significant predictive factor (hazard ratio [HR] = 1.40, 95% confidence interval [CI] = 1.03–1.91, *p* = 0.03) for deciding to stop treatment. A wife or husband (HR = 0.85, 95% CI = 0.72–1.02, *p* = 0.07) as well as children or brother/sister (HR = 0.77, 95% CI = 0.57–1.04, *p* = 0.08) provided a positive effect for continued treatment. Pharmacists are well positioned to play a role as an essential public health resource that can help improve adherence as well as social support that should be considered as an important component to improve adherence to long-term therapy in COPD as well as other chronic non-communicable diseases in low- and middle-income countries.

## Introduction

Chronic obstructive pulmonary disease (COPD), the most common chronic respiratory disease, is expected to become the third leading cause of death worldwide in 2020^1^. In 2015, 3.2 million people died from COPD worldwide, an increase of 11.6% compared with 1990. From 1990 to 2015, the prevalence of COPD increased by 44.2%^2^. The prevalence and morbidity of COPD increase sharply with age, and the burden of the disease is expected to grow over the next few decades as a result of an aging population and continued exposure to COPD risk factors^3^.

Treatment can improve COPD symptoms and reduce the frequency and severity of disease exacerbations. However, this improvement requires long-term recommended treatment regimens, and failure to achieve 80% adherence is a key barrier to achieving COPD treatment goals^1,4–6^. Multiple studies have shown that long-term adherence to chronic disease therapy is sub-optimal in real-world settings, and the World Health Organization (WHO) estimates that only 50% of patients prescribed such therapy are adherent to treatment^7^.

A recent study conducted in Slovenian patients with COPD found that slightly >53% were optimally adherent^8^. A study conducted in community pharmacies in the U.S. state of Missouri found that only 28.7% of participants were adherent (≥80% proportion of days covered)^9^. An observational study conducted in Turkey and Saudi Arabia found that 49.2% of participants had low adherence as measured by Morisky Medication Adherence Scale (MMAS-8), and a study in Hungary using this scale found that 58.2% of patients were optimally adherent^10,11^. A prospective cohort study of COPD patients in Italy found that adherence ranged from 35.5% to 79.1% using adherence to GOLD 2011 recommendations as the metric^12^.

Lack of adherence to COPD therapy has significant clinical, medico-economic, and societal impacts, including higher hospitalization rates, increased rates of severe exacerbations, reduced quality of life, increased emergency department visits, more days absent from work and more days of short-term disability, and employer costs of $1714 per year per non-adherent employee compared with adherent employees in a cross-sectional study conducted in United States^13^. In addition, good adherence to COPD therapy correlates with reduced healthcare resource utilization, including both direct healthcare costs and lost productivity^14^. Three-year mortality rates in non-adherent COPD patients are more than double than those in patients with good adherence^14^.

A key challenge in both assessing adherence rates to COPD therapy and evaluating methodologies to improve those rates is the lack of a single assessment approach^15,16^. A large variety of factors are associated with adherence to COPD therapy, including patient demographics; disease specifics and comorbidities; treatment regimen, satisfaction, and side effects; social and family support; cultural and socioeconomic factors; and level of health literacy^11,17,18^. In addition, poly-pharmacy, which may result from the need for multiple medications to manage comorbidities that often occur in COPD patients, is a common factor associated with poor adherence^19^. Health professionals have a critical role to play in helping patients manage multiple diseases and encouraging them to adhere to their treatment plans^18^.

While many factors that contribute to non-adherence to COPD therapy have been identified, the impact of social factors and the role of physicians and pharmacists on adherence have not been fully investigated in low- and middle-income countries. The significance of individual factors may also vary from country to country. Despite a high prevalence and burden of COPD in low- and middle-income countries, there are limited data related to COPD treatment adherence in these countries, including the potential role of physicians, pharmacists, and/or social support in patient adherence to COPD therapy. The goal of the current study was to identify factors associated with treatment adherence, especially the role of the physician, pharmacist, and social support, in making the decision to discontinue inhaled treatment in COPD patients in Egypt.

## Results

### Baseline characteristics of the study population

A total of 1311 patients were enrolled in the study between January 2017 and December 2018. The health professional population included 98 physicians and 205 pharmacists. The mean number of patients followed was 39.5 (standard deviation [SD] = 64.5, median [M] = 39.0) for physicians and 25.1 (SD = 24.0, M = 19.0) for pharmacists.

Of the 1311 patients included in the study, 631 (48.1%) of them stopped the treatment: 406 patients (64.3%) who stopped owing to physician decision, 170 (27.0%) who stopped owing to patient decision, and 55 patients (8.7%) who were deceased.

### Outcome measures

Patient support, adverse events, mean duration of treatment, and the number of patients attended by each participating physician and pharmacist were evaluated for the two comparison groups: patients in which the physician stopped treatment (*n* = 406) and patients stopped treatment due to their own decision (*n* = 170). As shown in Table 1, mean duration of treatment was similar between the two groups. In univariate analysis, significant differences between the two groups were found for wife or husband support, at least one treatment-related adverse event, and the number of patients attended by the pharmacist (Table 1).

**Table 1:** Comparison between patients for whom physician or patient made the decision to stop treatment and predictive factors of patient decision to stop treatment (univariate and multivariate analyses), Egypt 2017–2018 (*N* = 576).

	Physician decision (*n* = 406)	Patient decision (*n* = 170)	Univariate analysis			Multivariate analysis		
			HR	95% CI	*p*	HR	95% CI	*p*
Patient family support								
Himself	47.5	54.7	1.0			1.0		
Wife or husband	11.1	5.9	0.47	0.23–0.96	0.03	0.85	0.72–1.02	0.07
Children or brothers/sisters	41.4	39.4	0.81	0.55–1.18	0.34	0.77	0.57–1.04	0.08
Adverse event								
No	76.4	87.1	1.0			1.0		
At least one	23.6	12.9	0.49	0.30–0.80	0.003	0.85	0.69–1.05	0.85
Mean duration of treatment in months (SD, M, IQR)	3.8 (2.9, 3.3, 3.5)	3.7 (2.9, 2.9, 3.5)			0.54			
Number of patients attended by MD (quarter values)								
<16	24.0	20.7	1.0			1.0		
16–39	26.2	28.4	1.24	0.74–2.08	0.63	1.23	0.91–1.67	0.18
40–65	22.0	20.1	1.07	0.62–1.82	0.67	0.86	0.65–1.13	0.27
>65	27.8	30.8	1.29	0.78–2.14	0.45	1.03	0.81–1.31	0.83
Number of patients attended by pharmacist (quarter values)								
<6	24.4	16.5	1.0			1.0		
6–19	25.1	31.2	1.85	1.09–3.15	0.02	1.40	1.03–1.91	0.03
20–34	24.4	29.4	1.78	1.03–3.06	0.04	1.26	0.97–1.63	0.07
>34	26.1	22.9	1.28	0.72–2.25	0.79	1.05	0.83–1.34	0.68

*HR* hazard ratio, *CI* confidence interval, *SD* standard deviation, *M* median, *IQP* interquartile range, *MD* medical doctor.

**Fig. 1:**
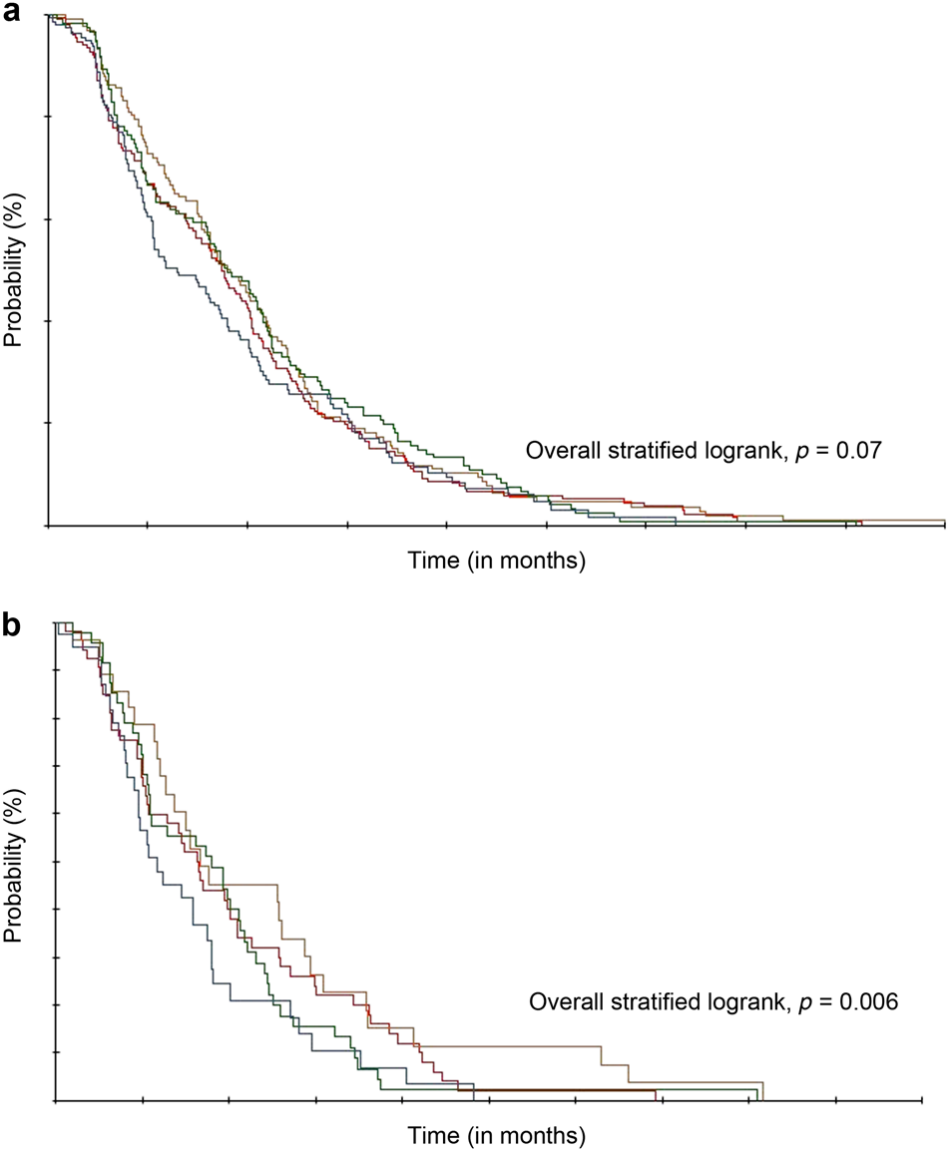
Kaplan–Meier analysis of treatment continuation as a function of the number of patients attended by the physician or the pharmacist. **a** Physician decision to continue treatment stratified on the number of patients attended by the physician. **b** Patient decision to continue treatment stratified on the number of patients attended by the pharmacist.

Figure 1 shows the probability of a patient remaining on treatment stratified by the number of patients attended by the physician (Fig. 1a) and the pharmacist (Fig. 1b). As shown in Fig. 1a, the probability of the physician making the decision to continue treatment was quite similar for the three first categories of patients attended by physician and was the lowest for patients attended the last quartile (*p* = 0.07). As shown in Fig. 1b, the probability of a patient making the decision to remain on treatment was significantly correlated with the number of patients attended by the pharmacist, with improved probability for patients whose pharmacist attended <6 patients and the probability decreasing proportionally with the number of patients attended by pharmacist (*p* = 0.006).

In a multivariate Cox proportional hazard model (Table 1), the quartile from 6 to 19 patients attended by the pharmacist was a significant predictive factor (hazard ratio [HR] = 1.40, 95% confidence interval [CI] = 1.03–1.91, *p* = 0.03) for the patient deciding to stop treatment. As shown in Table 1, multivariate analysis found that there was a positive independent association with family support and the patient choosing to remain on prescribed inhaled COPD therapy: wife or husband (HR = 0.85, 95% CI = 0.72–1.02, *p* = 0.07) and children or brothers/sisters (HR = 0.77, 95% CI = 0.57–1.04, *p* = 0.08).

## Discussion

This study was undertaken to identify factors predictive of COPD patients choosing to stop or remain on inhaled COPD therapy. The main results show that the number of patients attending a pharmacy has a significant effect on adherence to inhaled COPD therapy and that social support of the patient also has a positive impact on adherence.

Pharmacists may play a critical role in educational programs and disease management strategies that have the potential to improve adherence to therapy for chronic diseases. Pharmacists have a relevant role in the management of patients with COPD because of their medication and disease device expertise and frequent patient contact throughout all key stages of the patient care pathway (primary prevention, early detection/case finding, management and ongoing support, and review and follow-up)^20^. This contact enables pharmacists to provide an integrated COPD patient care pathway that includes advice and counseling related to patients’ medications, inhaler techniques (ITs), and the importance of adherence^20^. Consistent with this role and as suggested by the results reported, studies have shown that community pharmacists can identify patients with undiagnosed COPD and provide support for ongoing COPD care in a cost-effective manner^21^ and can have a positive effect on COPD management, especially with respect to IT education and treatment adherence^22^. A recent systematic review evaluating the impact of a community pharmacist-led intervention on patient medication adherence found that 61.5% of the 65 outcomes reported significantly favored the intervention group^23^. A very recent systematic review and meta-analysis has shown a positive impact of pharmacist-led interventions on inhalation technique and medication adherence in adult asthma and COPD patients^24^. In this study, significant improvement adherence was reported only among COPD patients. The findings suggest that pharmacist-led interventions based on the Information–Motivation–Behavioral skills model, a theoretical model use to promote health behavior^25^, may be more effective. Consequently, involving not only physicians but also pharmacists could be a relevant strategy for education of COPD patients in areas of low- and middle-income resources where physician demography is low.

Additional studies have shown increased COPD medication adherence, reduced risk of hospital admission, reduced health-related costs, and improved COPD control following pharmacist-based interventions^20,23,26^. A clinical pharmacist-based intervention conducted in COPD patients in India found a significant improvement in health-related quality of life in patients receiving the intervention compared with a control group^27^. This study also found a significant increase in medication adherence in the intervention group compared with controls^27^. A study conducted in Australia also found that a pharmacist-led intervention targeted to patients with hypertension, dyslipidemia, or depression saved US$1.9 billion annually^28^. This suggests that similar programs targeted to COPD patients might also yield health economic benefits.

In another context, a study of an access program for malaria treatment in five countries in sub-Saharan Africa also found that pharmacies play a critical role in providing access to quality-assured artemisinin combination therapy (ACT) for the treatment of malaria^29^. Further analysis of this program found that attending a pharmacy was a significant predictive factor for patients to purchase an ACT, the WHO-recommended treatment, and that patients attending a pharmacy as their first interaction with a healthcare provider were significantly more likely to purchase an ACT drug rather than a non-ACT drug in some countries^30^. These findings suggest also that pharmacies may play a significant role in accessing disease-appropriate therapies.

Taken together, these prior studies and the results of the current study emphasize that pharmacists have a critical role to play in improving multiple aspects of the care continuum for a variety of chronic and infectious diseases.

With respect to community pharmacists’ role in improving adherence to inhaled COPD therapy regimens, patients may benefit from the ongoing instruction in effective IT that pharmacists provide, as evidence suggests that IT declines over time^22^. One study of adherence to inhaled COPD therapy found that the only significant factor associated with overall significant mean adherence score was receiving repeated IT instruction from the patient’s respiratory physician^31^. This study also demonstrated significant and positive relationships between repeat IT instruction and adherence and health-related quality of life. Importantly, 18 of the 22 patients in this study who received repeat IT instruction received both verbal instructions and demonstration of effective IT, and none of the patients received written instructions. This suggests that direct interaction with a healthcare provider may be an important factor for ensuring that COPD patients maintain effective IT.

Although some national guidelines recommend that COPD patients receive IT instruction once each year^32^, the studies cited above suggest that more frequent IT training intervals may have a positive impact on adherence. Indeed, it is possible that the improved adherence observed among patients attending a pharmacy that sees fewer patients may be due to the pharmacist in these outlets having more time to spend with each patient on IT. Importantly, the amount of pharmacist time required to provide IT education and training must also be taken into account in designing interventions with the potential to improve adherence to inhaled COPD medication. A prior study evaluating the effectiveness of a community pharmacist–based home blood pressure monitoring program found that pharmacists required 100 min per patient to deliver the high-intensity intervention, which may not be practical, feasible, or cost-effective for many pharmacies on a walk-in basis^33^. This suggests that interventions requiring pharmacist time beyond a typical pharmacist–patient interaction might benefit from being implemented on an appointment basis rather than a walk-in basis^34^. Further studies specifically evaluating the role of the community pharmacist on patients’ IT and its impact on adherence and outcomes in COPD would be beneficial.

While the impact of time requirements indicates that larger pharmacies that have more pharmacists may be better able to support patient adherence to inhaled COPD therapy, the data from the current study suggest that smaller pharmacies have a more significant effect on this important aspect of care. The results of the current study indicate that the number of patients attended by the pharmacist significantly impacts patients’ decisions to discontinue COPD therapy. Given the important role of the pharmacist in helping COPD patients manage their disease, as described above, our data show that pharmacists may provide more strong effective support when they have fewer patients on which to focus. Consequently, strategies and interventions that allow pharmacists to manage smaller groups of patients or more effectively address the needs of larger patient groups could provide benefit with respect to patients’ decisions to discontinue treatment and should be evaluated. Further research work needs to be undertaken by policy makers to determine the approaches that can be used to motivate pharmacists to provide these services and to establish facilitators and barriers to allow successful implementation of pharmacist-based interventions in the future^21^.

While large and small pharmacies may influence treatment duration in some different disease areas, the findings presented here add to the data suggesting that pharmacists are ideally situated to help patients implement, modify, and sustain clearly defined, adherence-enhancing interventions in community settings and in the general population. In a world in which non-communicable diseases (NCDs) are strongly increasing and require chronic treatment that are increasingly costly, the role of the pharmacist should no longer be confined only to drug dispensation. With populations growing and aging, the demand on doctors, nurses, and other caregivers is such that, in most countries, they are no longer able to spend sufficient time educating patients on the need for adherence to treatment. Allowing pharmacists to play a more formal role in patient education and disease adherence may be critical to filling this gap and reducing healthcare systems’ ability to provide effective care.

The results of the current study also suggest that caregiver support may provide a protective benefit against patients deciding to discontinue COPD treatment. Our results show that a patient living alone, without social support, has an increased risk of deciding not to adhere to COPD treatment. A study of the influence of informal caregivers on adherence in COPD patients found that medication adherence was higher and the number of current smokers was lower among patients with a spouse or non-spousal caregiver compared with no caregiver^35^. The results of the current study are also supported by a recent analysis of social networks among patients with COPD, which found that most participants identified spouses as their most important social network member (adult children and grandchildren were also important to some participants) and that these individuals helped in organizing medications (i.e., packaging monthly supplies into shorter time periods, etc., administering medication, ordering medication, and monitoring medication supplies)^36^. Another study found that COPD patients who lived with others were more physically active than those who lived alone and that the chance of pulmonary rehabilitation participation was >11 times higher for patients who had a spouse or partner caregiver compared with patients without a caregiver^37^. Patient-related determinants of adherence to inhaled COPD medications found that patients identified having a caregiver or someone to help them remember, prepare, and purchase medication led to a good adherence behavior^38^. Caregiver support was also identified as beneficial in helping COPD patients in a variety of support domains, including managing symptoms and medication, adopting a healthy lifestyle, living positively with COPD, practical support, social and recreational life, and navigating healthcare services and encouragements^39^. These findings indicate that caregivers can help COPD patients meet their medication and rehabilitation goals and suggest that such support may also protect patients from deciding to discontinue COPD therapy.

Importantly, both caregiver support and adherence education are identified factors within the WHO’s five dimensions of adherence to long-term therapy^7^. While the current study confirms these factors as important for adherence in COPD therapy, additional study is needed to identify other factors within the WHO dimensions that may positively influence this critical aspect of patient care. True gains in improving adherence to COPD therapy and other long-term therapies will likely require integrated and holistic approaches that address all five of the WHO dimensions.

Strengths of this study include robust outcome measures that identified treatment status and the reason for treatment discontinuation (physician decision or patient decision). In addition, the study comprised a large patient sample size (*N* = 1311) and collected data from a very large sample of physicians and pharmacists. To our knowledge, this is one of the first studies to evaluate COPD adherence in a low-middle-income country, which is representative of the high COPD prevalence in these countries. This methodological approach is also a strength of this study, as it was a longitudinal study with prospective and regular follow-up of patients, physician-collected data, quality-validated data, and lack of missing data.

This study had several limitations. First, it did not include clinical data (i.e., severity of COPD) related to patients’ history of disease, COPD stage, previous and associated treatments, and lung function. It is well known that these factors may affect the prognosis of COPD. With respect to the reason for discontinuation, we used a binary categorization schema of discontinuation per patient decision or per physician decision. Although this does not provide insight into the underlying reason why a patient or physician discontinued treatment, the information collected as a binary variable could minimize a potential information bias.

Second, the study used a patient’s decision to discontinue treatment as a proxy measure for adherence, which may be considered as lacking precision and biasing the treatment outcome. However, it provides a robust and conservative estimate of adherence and is a consistent indicator across all groups. The ability to capture more precise information about the exact reason for patient non-adherence should improve our understanding of the clinical, social, cultural, and other factors that impact adherence to inhaled therapy in patients with COPD and other long-term treatments as suggested in the WHO five dimensions of adherence^7^. Third, some factors may also have been overlooked owing to insufficient statistical power. However, despite these limitations, several variables showed a trend that approached or reached statistical significance, identifying potentially interesting factors associated with non-adherence that could warrant further evaluation. Finally, the adherence scale used in the current study is not validated, unlike the Morisky self-reported adherence scale used in other studies^10,11^. However, we believe that the use of the physician’s report on the decision to stop or not report treatment is an appropriate and robust proxy for adherence measure.

In low- and middle-income countries, NCDs are placing a growing burden on individuals, societies, and economies. The lessons learned from this study in COPD may provide important insight into our growing understanding of barriers to effective NCDs management and strategies for eliminating these barriers. Pharmacists are well positioned to play a role as an essential public health resource that can help improve adherence, which is critical for improving healthcare outcomes. In the current study, the number of patients attended by a pharmacist had a significant effect on patients’ decisions to discontinue therapy, with patients more likely to decide to discontinue when pharmacists were attending to larger numbers of patients. Reducing pharmacist caseloads or increasing their ability to provide disease management services could provide both clinical and economic benefit. Pharmacist involvement could play a pivotal role in increasing adherence to chronic disease therapies, especially in low- and middle-income countries. In these countries, involving health professionals other than the treating physician, such as the pharmacist, could be an efficient strategy because it accounts for physicians’ workloads and their limited time available for patient education and counseling as well lack of availability of medical doctors in rural and remote areas. These findings also underscore the need to consider the role that pharmacists may play in the continuum of chronic disease therapy beyond simply dispensing medications and how pharmacists may be optimally leveraged within the WHO’s five dimensions of adherence.

The results presented here warrant further study in order to more fully elucidate the role of pharmacists and social support in COPD treatment adherence specifically and within chronic NCDs more broadly. These results should also help to inform the design, evaluation, and implementation of pharmacist- and caregiver-based interventions to improve outcomes in patients with chronic NCD adherence that have already been established^7^.

## Methods

### Study design

A prospective cohort study was conducted in 2017–2018 in Cairo (Egypt). In 2017, Axios International implemented a new drug access program in Egypt for COPD patients treated with an inhaled medication in a context of improvement of access to drug program. Volunteer physicians were invited by Axios to join the program and it was implemented through a network of physicians in public and private healthcare centers. Physicians were initially approached for participation in the study through an introductory letter, followed by an in-person appointment. In a context of evaluation of the program, Axios International conducted the survey using an established tracing strategy for a pharmaceutical product. The manufacturer of this product had no role in the study design, analyses, or drafting of the manuscript.

### Study population

Inclusion criteria were the following: patients who were aged >18 years, who had a physician-confirmed diagnosis of COPD and a physician prescription for a medication inhaler, and who were willing to undergo the prescribed treatment. Patients were enrolled from the public and private sectors so as to reflect as well as possible the representativeness of distribution of public and private treatment patterns in the general population. Participating patients received medication and care at multiple public and private hospitals and health facilities in Cairo. Participating subjects paid for a certain number of treatment cycles and received a portion of treatment free of charge based on their ability to pay for treatment as assessed using the validated Patient Financial Eligibility Tool^40^. All information from patient files was collected anonymously from medical reports, so no written informed consent of patient was collected. No personal identifying information was also collected. The Ethical Committee of Rouen University Hospital (CERDE-HLJ) approved the research (#E2019-65).

### Data collection

Patients in the study attended their own treating physicians who performed COPD clinical assessments consistent with local medical practices and managed treatment guidelines. Physicians administered a questionnaire to participating patients at monthly visits and at the end of each visit; treatment status was binary categorized as treatment continued or treatment stopped. The questionnaire also collected the name and the address of the pharmacy from which the patients purchased their prescribed inhaler medication. The physician also collected data regarding patient deaths and treatment-related adverse events. Data regarding patients’ social support related to assistance taking daily COPD treatment was collected as the absence of social support for the patient or patient supported by spouse, children, and siblings.

The study endpoint was adherence to inhaled COPD therapy at 1 year following initiation of treatment. If the status was categorized as treatment stopped, the reason for discontinuation was categorized as per patient decision or per physician decision. Patients who had decided to stop treatment were considered non-adherent to COPD therapy for the purposes of estimating adherence rates within the study population.

### Statistical analysis

Descriptive statistics were computed for the characteristics of patients included in the program and are calculated as mean and SD, M, and interquartile range for quantitative variables and as percentage for qualitative variables. Chi-square test was used for qualitative data comparisons; Student’s *t* test for quantitative comparisons.

Quartile values were established for the number of patients followed by each physician in the study and the number of patients purchasing their inhaled COPD therapy at each pharmacy.

Kaplan–Meier method was used to obtain the probability of treatment continued or stopped based on patient or physician decision. Kaplan–Meier plots were stratified on the number of patients attended by the physician and by the pharmacist. Differences among the quartiles were tested using stratified logrank tests. Multivariate Cox proportional-hazard models were fitted to identify predictive factors of the patients’ decision to stop treatment. All variables tested in univariate analysis were included in the Cox model. For all analyses, a significance level of 0.05 was used. Statistical analyses were carried out in Statview^(R)^ 5.0 SAS (SAS Institute Inc.).

### Ethics statement

Participation in this study was voluntary, with all the physicians included. All information from patient files was collected anonymously from medical reports, so no written informed consent was collected. No personal identifying information was collected. The Ethical Committee of Rouen University Hospital (CERDE-HLJ) approved the research (#E2019-65).

### Reporting summary

Further information on experimental design is available in the Nature Research Reporting Summary linked to this paper.

## Supplementary information

Reporting summary

## Data Availability

Full datasets are not publicly available to protect anonymity of physicians and theirs patients and to ensure data security.

## References

[1] Global Initiative for Chronic Obstructive Lung Disease. Global strategy for the diagnosis, management, and prevention of chronic obstructive pulmonary disease (2019 report). https://goldcopd.org/wp-content/uploads/2018/11/GOLD-2019-v1.7-FINAL-14Nov2018-WMS.pdf (2019). Accessed 4 Feb 2020.

[2] GBD 2015 Chronic Respiratory Disease Collaborators. (2017). Global, regional, and national deaths, prevalence, disability-adjusted life years, and years lived with disability for chronic obstructive pulmonary disease and asthma, 1990-2015: a systematic analysis for the Global Burden of Disease Study 2015. Lancet Respir. Med..

[3] Mathers CD, Loncar D (2006). Projections of global mortality and burden of disease from 2002 to 2030. PLoS Med..

[4] Haahtela T (2009). Thirteen-year follow-up of early intervention with an inhaled corticosteroid in patients with asthma. J. Allergy Clin. Immunol..

[5] Cote C (2005). Pharmacoeconomics and the burden of COPD. Clin. Pulm. Med..

[6] Global Initiative for Asthma. Global strategy for asthma management and prevention. https://ginasthma.org/wp-content/uploads/2019/01/2011-GINA.pdf (2011). Accessed 4 Feb 2020.

[7] World Health Organization. Adherence to long-term therapies: evidence for action. https://www.who.int/chp/knowledge/publications/adherence_introduction.pdf?ua=1 (2003). Accessed 4 Feb 2020.

[8] Horvat N, Locatelli I, Kos M, Janezic A (2018). Medication adherence and health-related quality of life among patients with chronic obstructive pulmonary disease. Acta Pharmacol..

[9] Bollmeier SG (2019). Assessment of symptom burden and adherence to respiratory medications in individuals self-reporting a diagnosis of COPD within a community pharmacy setting. J. Am. Pharm. Assoc. (2003).

[10] Kokturk N (2018). Adherence to COPD treatment in Turkey and Saudi Arabia: results of the ADCARE study. Int. J. Chron. Obstruct. Pulmon. Dis..

[11] Agh T, Inotai A, Meszaros A (2011). Factors associated with medication adherence in patients with chronic obstructive pulmonary disease. Respiration.

[12] Scalone G (2018). Pharmacological approach and adherence to treatment recommendations in frequently and non-frequently exacerbating COPD patients from Italy: MISTRAL - the prospective cohort, observational study. Pulm. Pharmacol. Ther..

[13] van Boven JF (2014). Clinical and economic impact of non-adherence in COPD: a systematic review. Respir. Med..

[14] Makela MJ, Backer V, Hedegaard M, Larsson K (2013). Adherence to inhaled therapies, health outcomes and costs in patients with asthma and COPD. Respir. Med..

[15] Gossec L, Tubach F, Dougados M, Ravaud P (2007). Reporting of adherence to medication in recent randomized controlled trials of 6 chronic diseases: a systematic literature review. Am. J. Med. Sci..

[16] Boland MR (2016). Investigating the association between medication adherence and health-related quality of life in COPD: methodological challenges when using a proxy measure of adherence. Respir. Med..

[17] Lopez-Campos JL, Quintana Gallego E, Carrasco Hernandez L (2019). Status of and strategies for improving adherence to COPD treatment. Int. J. Chron. Obstruct. Pulmon. Dis..

[18] Sanduzzi A (2014). COPD: adherence to therapy. Multidiscip. Respir. Med..

[19] Barnestein-Fonseca P (2011). Efficacy and safety of a multifactor intervention to improve therapeutic adherence in patients with chronic obstructive pulmonary disease (COPD): protocol for the ICEPOC study. Trials.

[20] van der Molen T, van Boven JF, Maguire T, Goyal P, Altman P (2017). Optimizing identification and management of COPD patients - reviewing the role of the community pharmacist. Br. J. Clin. Pharmacol..

[21] Twigg MJ, Wright DJ (2017). Community pharmacy COPD services: what do researchers and policy makers need to know?. Integr. Pharm. Res. Pract..

[22] Hesso I, Gebara SN, Kayyali R (2016). Impact of community pharmacists in COPD management: inhalation technique and medication adherence. Respir. Med..

[23] Milosavljevic A, Aspden T, Harrison J (2018). Community pharmacist-led interventions and their impact on patients’ medication adherence and other health outcomes: a systematic review. Int. J. Pharm. Pract..

[24] Jia X (2020). Effect of pharmacist-led interventions on medication adherence and inhalation technique in adult patients with asthma or COPD: a systematic review and meta-analysis. J. Clin. Pharm. Ther..

[25] Fisher JD, Fisher WA, Amico KR, Harman JJ (2006). An information-motivation-behavioral skills model of adherence to antiretroviral therapy. Health Psychol..

[26] Zhong H, Ni XJ, Cui M, Liu XY (2014). Evaluation of pharmacist care for patients with chronic obstructive pulmonary disease: a systematic review and meta-analysis. Int. J. Clin. Pharm..

[27] Suhaj A, Manu MK, Unnikrishnan MK, Vijayanarayana K, Mallikarjuna Rao C (2016). Effectiveness of clinical pharmacist intervention on health-related quality of life in chronic obstructive pulmonary disorder patients - a randomized controlled study. J. Clin. Pharm. Ther..

[28] Cutler RL (2019). Pharmacist-led medication non-adherence intervention: reducing the economic burden placed on the Australian health care system. Patient Prefer Adherence.

[29] Davis B (2013). Artemisinin-based combination therapy availability and use in the private sector of five AMFm phase 1 countries. Malar. J..

[30] Ladner J, Davis B, Audureau E, Saba J (2017). Treatment-seeking patterns for malaria in pharmacies in five sub-Saharan African countries. Malar. J..

[31] Takemura M (2011). Relationships between repeated instruction on inhalation therapy, medication adherence, and health status in chronic obstructive pulmonary disease. Int. J. Chron. Obstruct. Pulmon. Dis..

[32] National Clinical Guideline Centre (UK). *Chronic Obstructive Pulmonary Disease: Management of Chronic Obstructive Pulmonary Disesae in Adults and in Primary and Secondary Care* (Royal College of Physicians (UK), London, 2010). 22319804

[33] Zillich AJ, Sutherland JM, Kumbera PA, Carter BL (2005). Hypertension outcomes through blood pressure monitoring and evaluation by pharmacists (HOME study). J. Gen. Intern. Med..

[34] Chabot I, Moisan J, Gregoire JP, Milot A (2003). Pharmacist intervention program for control of hypertension. Ann. Pharmacother..

[35] Trivedi RB, Bryson CL, Udris E, Au DH (2012). The influence of informal caregivers on adherence in COPD patients. Ann. Behav. Med..

[36] Schafheutle EI, Fegan T, Ashcroft DM (2018). Exploring medicines management by COPD patients and their social networks after hospital discharge. Int. J. Clin. Pharm..

[37] Chen Z, Fan VS, Belza B, Pike K, Nguyen HQ (2017). Association between social support and self-care behaviors in adults with chronic obstructive pulmonary disease. Ann. Am. Thorac. Soc..

[38] Duarte-de-Araujo A, Teixeira P, Hespanhol V, Correia-de-Sousa J (2018). COPD: understanding patients’ adherence to inhaled medications. Int. J. Chron. Obstruct. Pulmon. Dis..

[39] Gardener AC, Ewing G, Kuhn I, Farquhar M (2018). Support needs of patients with COPD: a systematic literature search and narrative review. Int. J. Chron. Obstruct. Pulmon. Dis..

[40] Audureau E (2019). Ability to pay for medication: a clustering analysis of 1404 patients with the Patient Financial Eligibility Tool. J. Comp. Eff. Res..

